# Comparative clinical outcomes of dual cannulated screw-cable system vs. Kirschner wire-cable fixation in type C patellar fractures

**DOI:** 10.3389/fsurg.2025.1594907

**Published:** 2025-09-01

**Authors:** Huan Yang, Yusong Yuan, Lei Shi, Fangda Si, Jiaqi Liu, Ying Chen, Xiaodong Xu

**Affiliations:** Orthopedic Trauma, China-Japan Friendship Hospital, Beijing, China

**Keywords:** patellar fracture, dual cannulated screw-cable system, Kirschner wire-cable fixation, functional outcomes, hardware irritation

## Abstract

**Introduction:**

To compare the clinical efficacy and safety of the dual cannulated screw-cable (DCSC) system with those of conventional Kirschner wire-cable (KWC) fixation in the management of patellar fractures. Traditional KWC fixation, while widely used since the 1970s, is associated with high complication rates, including symptomatic hardware irritation (up to 42%) and loss of reduction (12%–15% in transverse fractures), due to its biomechanical limitations such as lack of interfragmentary compression and prominent hardware causing soft tissue irritation. The DCSC system, introduced as a promising alternative, offers active interfragmentary compression and reduced soft tissue irritation, potentially addressing these limitations. However, few clinical studies have directly compared the outcomes of DCSC and KWC fixation in patellar fractures. This study aims to fill this gap by evaluating functional recovery, radiographic union, and complication rates between the two fixation methods.

**Methods:**

This retrospective cohort study included 127 patients with patellar fractures (AO/OTA 34-C) treated between January 2020 and December 2023. The patients were stratified into DCSC (*n* = 26) and KWC (*n* = 101) groups. The primary outcomes included functional recovery (Lysholm and Böstman scores) at 3 and 12 months postoperatively. The secondary outcomes included radiographic union time, complication rates, and reoperation rates. Between-group comparisons were performed using t tests and chi-square tests (*p* < 0.05).

**Results:**

The DCSC group demonstrated superior short-term functional outcomes, with significantly higher Lysholm scores at 3 months (76.0 ± 6.1 vs. 70.4 ± 2.9, *p* < 0.001) and significantly higher Böstman scores across all fracture classifications (C1: 21.5 vs. 17.5; C2: 21.6 vs. 17.2; C3: 21.3 vs. 17.6; all *p* < 0.001). Notably, C2 fractures treated with DCSC exhibited the greatest improvement in Lysholm scores (at 3 months, *p* < 0.001). DCSC also resulted in shorter operative times (62.9 ± 1.8 vs. 76.0 ± 1.4 min, *p* < 0.001) and reduced symptomatic hardware irritation (3.8% vs. 21.8%, *p* = 0.03). Radiographic union was faster in the DCSC group (3.04 vs. 3.50 months, *p* < 0.001). However, the Lysholm and Böstman scores at 12 months were similar between the groups (*p* > 0.05), and the reoperation rates at 12 months were comparable (3.8% vs. 2.0%, *p* = 0.82).

**Conclusion:**

Compared with KWC fixation, the DCSC system provides superior early functional recovery, fewer complications, and faster fracture healing, particularly in complex intra-articular fractures (OTA 34-C2). However, the benefits of the DCSC system in simpler or more comminuted fracture (C1/C3) diminish over time, and caution is warranted when using this system in comminuted or distal coronal plane fractures owing to potential compression limitations. These findings support the use of DCSC as a first-line option for C2 fractures, although long-term studies are needed to assess implant durability.

## 1 Introduction

Patellar fractures, which account for approximately 1% of all skeletal injuries, pose significant challenges in the context of orthopedic trauma because of their impact on the integrity of the knee extensor mechanism (1). These fractures predominantly affect active individuals aged 20–50 years, and they are often caused by high-energy trauma, with long-term disability rates exceeding 25% when inadequately treated (2). Traditional fixation methods, such as Kirschner wire-cable (KWC) fixation, have been widely used since the 1970s. However, KWC fixation is associated with high complication rates, including symptomatic hardware irritation (reported in up to 42% of cases) and loss of reduction (occurring in 12%–15% of transverse fractures) (3, 4). These limitations underscore the need for more effective surgical techniques.

The dual cannulated screw-cable (DCSC) system has emerged as a promising alternative, as it offers biomechanical advantages such as active interfragmentary compression and reduced soft tissue irritation (5, 6). Finite element analyses have demonstrated that cannulated screws generate 18%–23% greater compression force than K-wires under cyclic loading, thereby potentially addressing the primary failure modes of traditional tension band constructs (6). Despite these theoretical benefits, few clinical studies have directly compared the outcomes of DCSC and KWC fixation in patellar fractures.

Therefore, the primary objective of this retrospective cohort study was to compare the clinical efficacy, complication rates, and knee function recovery between DCSC and KWC fixation in the treatment of patellar fractures. By evaluating functional outcomes, radiographic union time (Union required bridging of ≥3 cortices on AP and lateral radiographs.), and reoperation rates, we aim to provide evidence-based recommendations for surgical strategy selection in patellar fracture management.

## 2 Materials and methods

### 2.1 Study design

**Figure 1:**
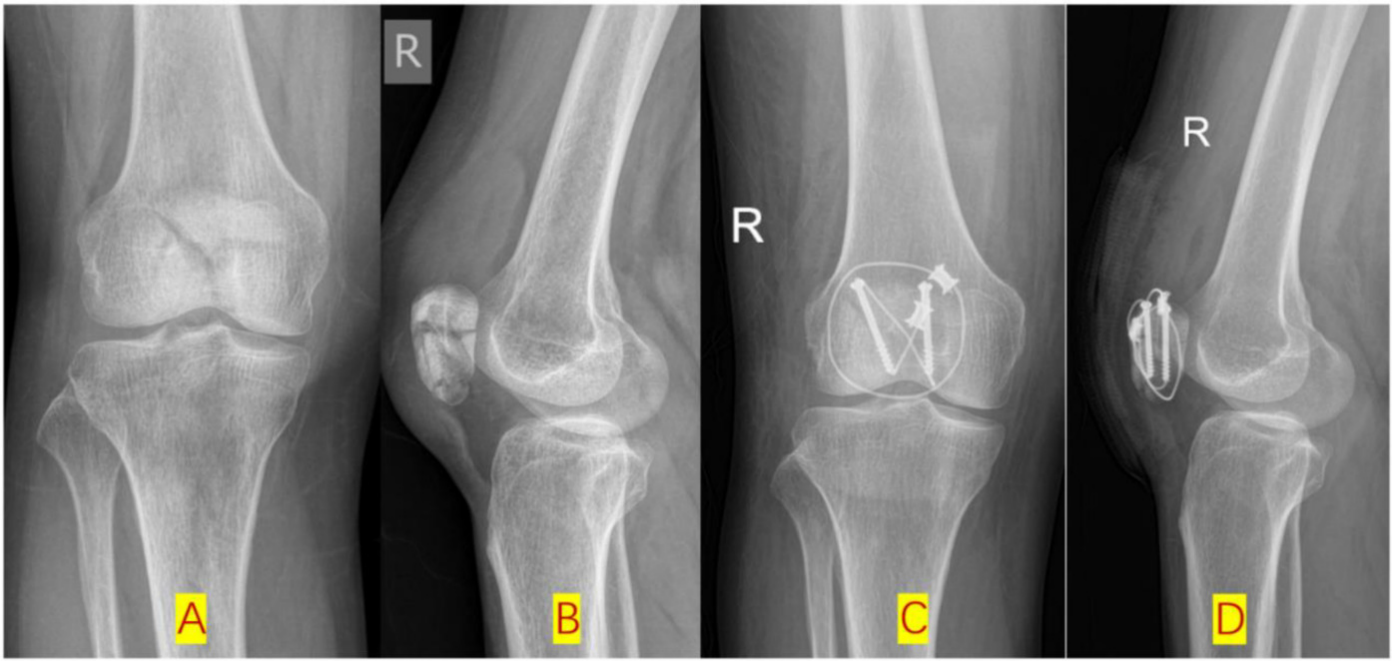
Additional cerclage wiring was occasionally used to stabilize fragments in C3 group before or after DCSC application. **(A,B)**: Pre-op x-ray posteroanterior (PA) and lateral radiographic views; **(C,D)**: post-op x-ray PA and lateral radiographic views.

This retrospective cohort study was conducted in our orthopedic trauma department (single institute) and included patients treated for patellar fractures between January 2020 and December 2023. The study was conducted in accordance with the ethical standards of the Institutional Review Board of China-Japan Friendship Hospital and the Helsinki Declaration. The study protocol was approved by the Institutional Review Board (IRB No. 2023-KY-337-1), and informed consent was waived for this retrospective study by the Institutional Review Board. Consent to Participate declarations: not applicable. Fractures were classified according to the AO/OTA system (1, 7) as follows: 34-C1: Simple articular fracture (e.g., transverse fracture). 34-C2: Simple articular fracture with metaphyseal comminution. 34-C3: Comminuted articular fracture. The DCSC system used in our study employed 4.0-mm diameter, half-threaded, equidistant-pitch cannulated screws (Johnson & Johnson) to optimize interfragmentary compression. To minimize soft tissue irritation, the DCSC technique included: Countersinking screw heads: the screw heads were positioned in immediate proximity to the patellar surface. Trimming cable ends: flush with the screws and embedding them in the quadriceps tendon and the patellar tendon. Intraoperative fluoroscopy to confirm hardware positioning. For severely comminuted C3 fractures, additional mini-fragment screws or cerclage wiring was occasionally used to stabilize small fragments before or after DCSC application. This adjunctive technique was noted in 5/26 DCSC cases (Figure 1).

The inclusion criteria were as follows: (1) adult patients (≥18 years) with OTA 34-A, B, and C type patellar fractures (8); (2) surgical treatment with either DCSC or KWC fixation; and (3) minimum follow-up of 12 months. The exclusion criteria were as follows: (1) open fractures or pathological fractures; (2) age <18 years; or (3) incomplete clinical or radiographic data (Supplementary Table 1, Supplementary Figure 1).

### 2.2 Patient groups and surgical techniques

**Figure 2:**
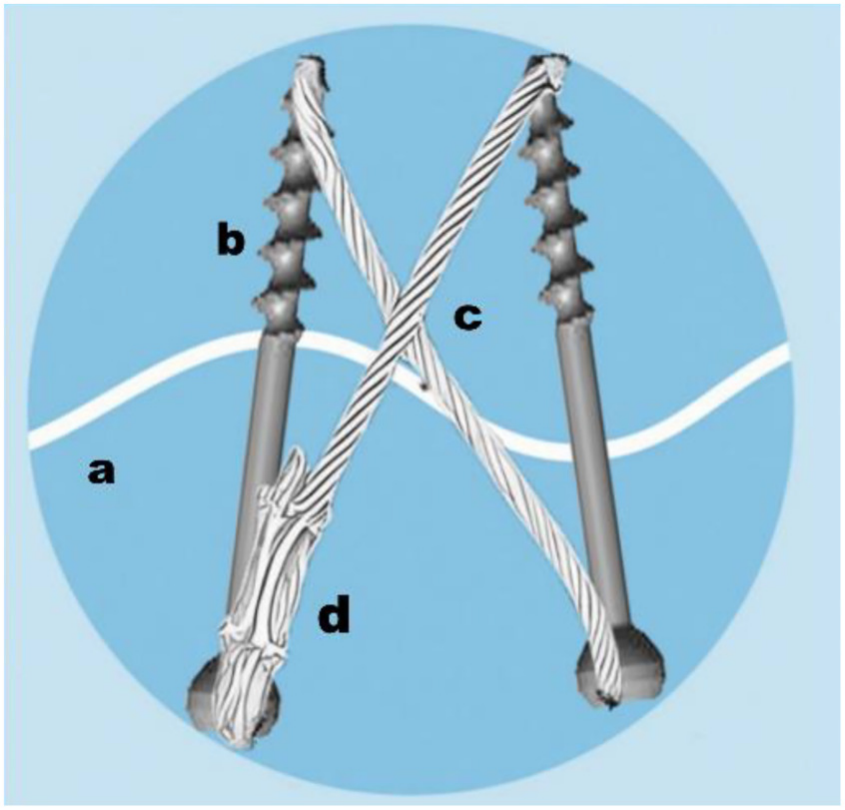
Schematic diagram of the DCSC system in fixing patellar fracture. (**a:** fracture line; **b:** cannulated screw; **c:** 1.0 mm titanium cable; **d:** cable clamp).

**Figure 3:**
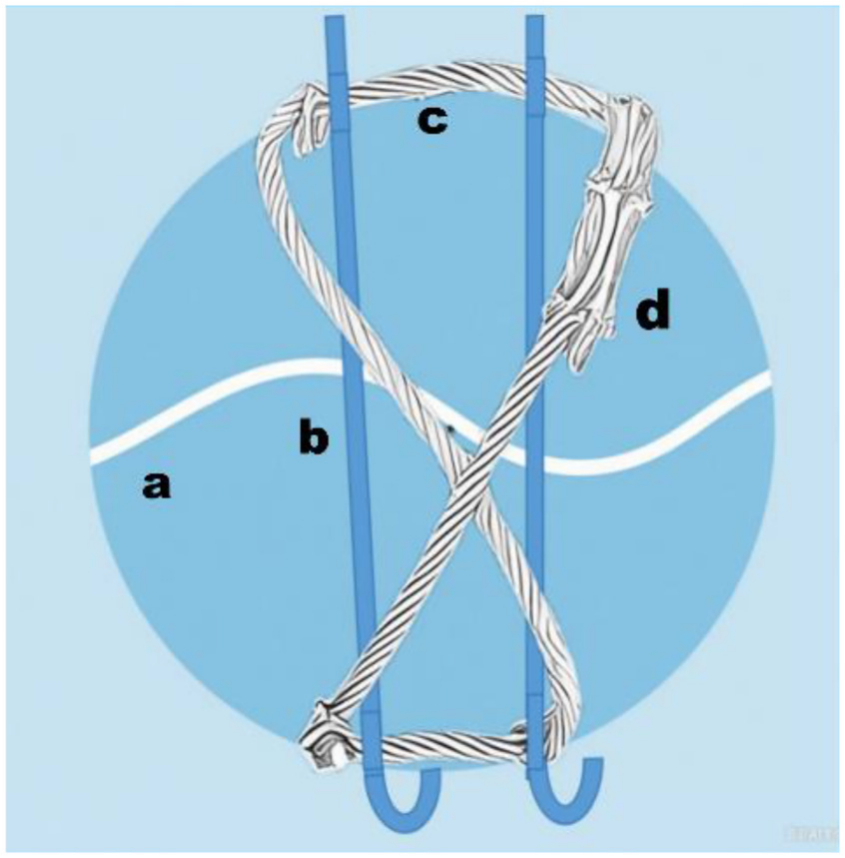
schematic diagram of the KWC system in fixing patellar fracture. [**a:** fracture line; **b:** Kirschner wires (K-wires): 2.0 mm diameter, stainless steel; **c** 1.0 mm titanium cable; **d:** cable clamp].

Patients were divided into two groups based on the fixation method: in the DCSC group, patients were treated with dual cannulated screws combined with cable fixation, performed by two experienced surgeons within the department who were proficient in the DCSC technique; in the KWC group, patients were treated with traditional Kirschner wire-cable tension band fixation, performed by other surgeons in the department who were skilled in the KWC method but did not have experience with the DCSC system. KWC Group: Kirschner wires (K-wires): 2.0 mm diameter, stainless steel, inserted longitudinally. Cerclage wiring: 1.0 mm titanium cable in a figure-of-eight configuration and/or tensioned circumferentially occasionally. DCSC Group: Cannulated screws: 4.0 mm diameter, titanium, half threaded, equidistant pitch. Cerclage wiring: 1.0 mm titanium cable passed through the screws in a figure-of-eight configuration and/or tensioned circumferentially occasionally (Figures 2, 3).

### 2.3 Data collection

The following data were extracted from electronic medical records: baseline characteristics, including age, sex, and mechanism of injury; fracture classification according to the AO/OTA system (34- C1, C2 or C3) (1); and surgical parameters, including operative time (minutes), Fluoroscopy usage was quantified as number of intraoperative shots. (Number of intraoperative C-arm images), high-energy injury proportion (%) and length of hospital stay (days). Both groups followed the same post-operation protocol: x-ray for the first month post-surgery. Monthly until union, then at 6 and 12 months. Functional outcomes were assessed using the validated Lysholm Knee Score (8), which evaluates symptoms (pain, instability, swelling, locking, limp, stair climbing) and functional limitations (squatting, need for support) on a scale of 0–100 (higher scores indicating better function). The Böstman Patellofemoral Score (2) was also used, specifically designed for patellar fractures; it assesses active range of motion (0–6 points), pain (0–6 points), and ability to work (0–6 points) on a scale of 0–18 (higher scores indicating better function). Assessments were performed by independent physiotherapists blinded to the fixation method. No missing data occurred in primary outcomes (Lysholm/Böstman scores), complications, or reoperation rates, as all 127 patients completed the 12-month follow-up. Baseline variables (e.g., age, sex, fracture type) were fully documented in electronic medical records.

#### 2.3.1 Primary outcomes

The primary outcomes were functional outcomes, which were assessed using the Lysholm Knee Score and the Böstman scale at 3 and 12 months postoperatively (2, 8). Additionally, subgroup analyses were conducted to compare the Lysholm Knee Score and the Böstman scale across different fracture classifications (C1, C2, and C3) to evaluate the efficacy of the fixation methods in specific fracture patterns.

#### 2.3.2 Secondary outcomes

The secondary outcomes were as follows: radiographic union time, bridging callus formation standard defined as visible bridging trabeculae across ≥3 cortices on lateral radiographic and posteroanterior (PA) views (7). Cortices are assessed at the fracture site, with trabecular continuity indicating biological healing. This aligns with AO Foundation guidelines for fracture healing assessment (AO/OTA classification system). Fracture Line Obliteration: Progressive disappearance of the fracture line on serial radiographs, with no visible gap at the fracture interface (1). Clinical correlation: union must correlate with functional recovery (e.g., pain-free weight-bearing, improved Lysholm/Böstman scores) (2, 8) to confirm clinical healing (8). Intraoperative fluoroscopy usage: Quantified as the number of C-arm shot images acquired during surgery. Postoperative complications, including symptomatic hardware irritation, superficial infection, and nonunion; and the reoperation rate, defined as the need for hardware removal or revision surgery.

### 2.4 Statistical analysis

Continuous variables are expressed as the means ± standard deviations (SDs) and were compared using Student's t test or the Mann‒Whitney U test, as appropriate. Categorical variables are presented as frequencies (%) and were compared using the chi-square test or Fisher's exact test. A *p* value <0.05 was considered to indicate statistical significance. All analyses were performed using SPSS version 21.0 (IBM Corp., Armonk, NY, USA). Given the retrospective design, no formal *a priori* power calculation was performed. However, *post hoc* power analyses for primary outcomes (Lysholm/Böstman scores at 3 months) confirmed adequate power (>80%) to detect statistically significant differences. To address potential selection bias, we performed propensity score matching (PSM) using a 1:1 nearest-neighbor algorithm with a caliper width of 0.2 standard deviations. Covariates included age, sex, fracture type (OTA 34-C1/C2/C3). After matching, 26 DCSC patients were paired with 26 KWC patients. Balance was assessed using standardized mean differences (SMD), where SMD <0.1 indicated adequate balance (Table 4). All outcomes were re-analyzed in the matched cohort.

## 3 Results

### 3.1 Baseline characteristics

A total of 127 patients were included in the study, including 26 in the DCSC group and 101 in the KWC group. The baseline demographic and fracture characteristics were comparable between the two groups (Table 1). There were no significant differences in age (mean: 62.6 ± 13.5 vs. 60.0 ± 13.0 years, *p* = 0.36), sex distribution (male: 20.5% vs. 79.5%, *p* = 0.52), or fracture type (OTA34-C1: 19.6% vs. 80.4%; 34-C2: 22.2% vs. 77.8%; 34-C3: 19.4% vs. 80.6%, *p* = 0.94) between the groups. There were also no significant differences in High-energy injury proportion (18.6% vs. 21.4%, *p* = 0.71) or length of hospital stay (6.9 ± 1.8 vs. 7.6 ± 2.8, *p* = 0.22).

**Table 1:** Demographic and clinical characteristics of the DCSC and KWC groups.

Variable	DCSC group (*n* = 26)	KWC group (*n* = 101)	*p*
Age (years)	62.6 ± 13.5	60.0 ± 13.0	0.36
Sex (male)	20.5%	79.5%	0.52
Fracture type			
- OTA 34-C1	19.6%	80.4%	
-C2	22.2%	77.8%	0.94
-C3	19.4%	80.6%	
High-energy injury proportion	18.6%	21.4%	0.71
Hospital stay (days)	6.9 ± 1.8	7.6 ± 2.8	0.22

### 3.2 Clinical outcomes

**Figure 4:**
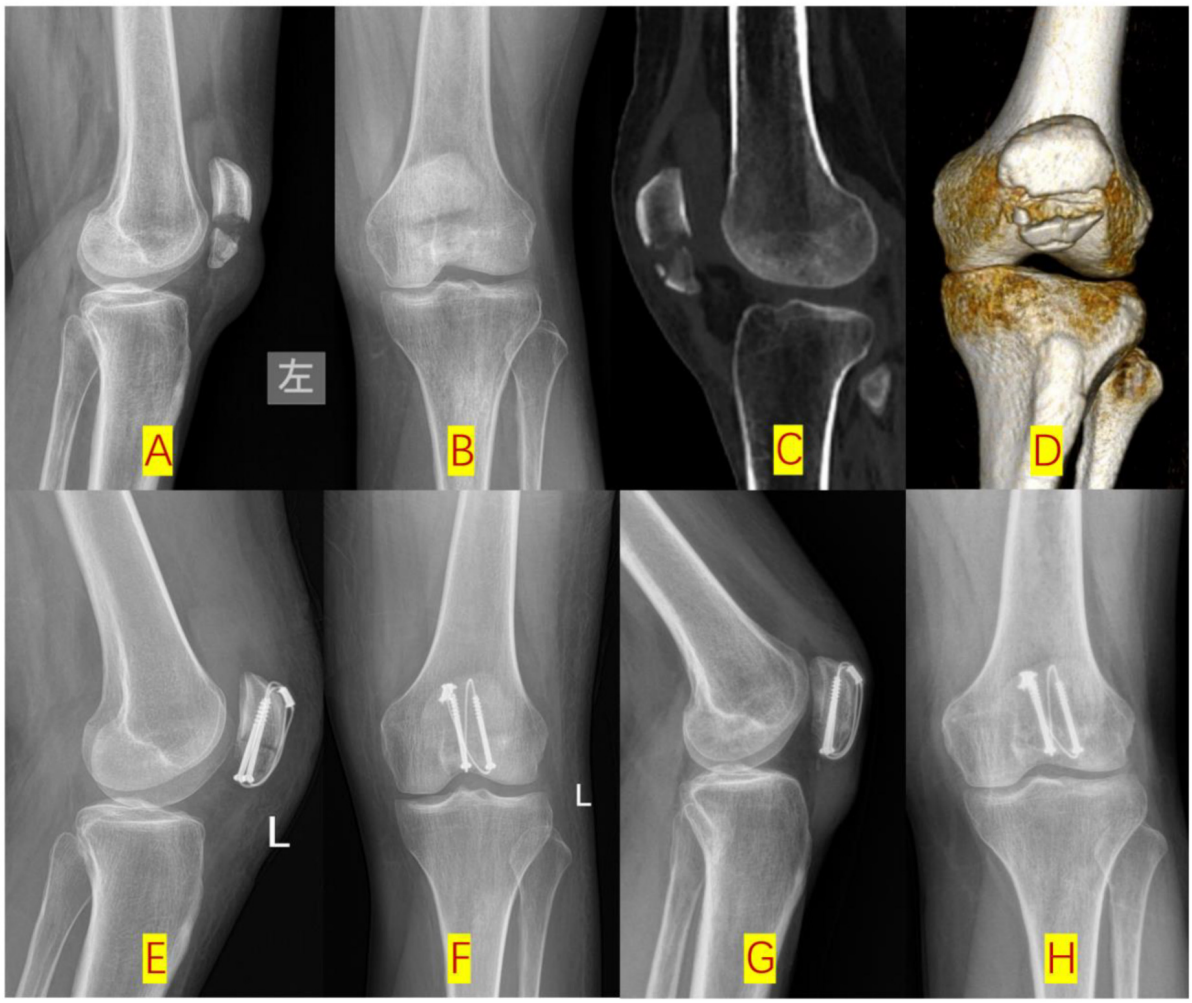
Type-C2 patellar fracture fixed by dual cannulated screw-cable system (DCSC) **(A,B)**: Pre-op x-ray lateral radiographic and posteroanterior (PA) views; **(C,D)**: Pre-op CT sagittal and 3D reconstructions; **(E,F)**: post-op lateral and PA views; **(G,H)**: 3-month post-op follow-up lateral and PA views.

Functional scores: Compared with the KWC group, the DCSC group exhibited statistically significant improvements in Lysholm scores (76.0 ± 6.1 vs. 70.4 ± 2.9, *p* < 0.001) and Böstman scores (21.5 ± 0.5 vs. 17.5 ± 0.5, *p* < 0.001) at the 3-month postoperative follow-up, indicating the superior short-term functional recovery of the DCSC system. However, this initial advantage diminished over time, as no significant differences in the Lysholm scores (88.5 ± 6.4 vs. 90.3 ± 6.0, *p* = 0.17) or Böstman scores (22.0 ± 0.8 vs. 21.9 ± 0.8, *p* = 0.79) were observed between the two groups at the 12-month postoperative assessment, thus suggesting convergence in long-term outcomes. Radiographic union was faster in the DCSC group (3.04 vs. 3.50 months, *p* < 0.001) (Figure 4).

**Figure 5:**
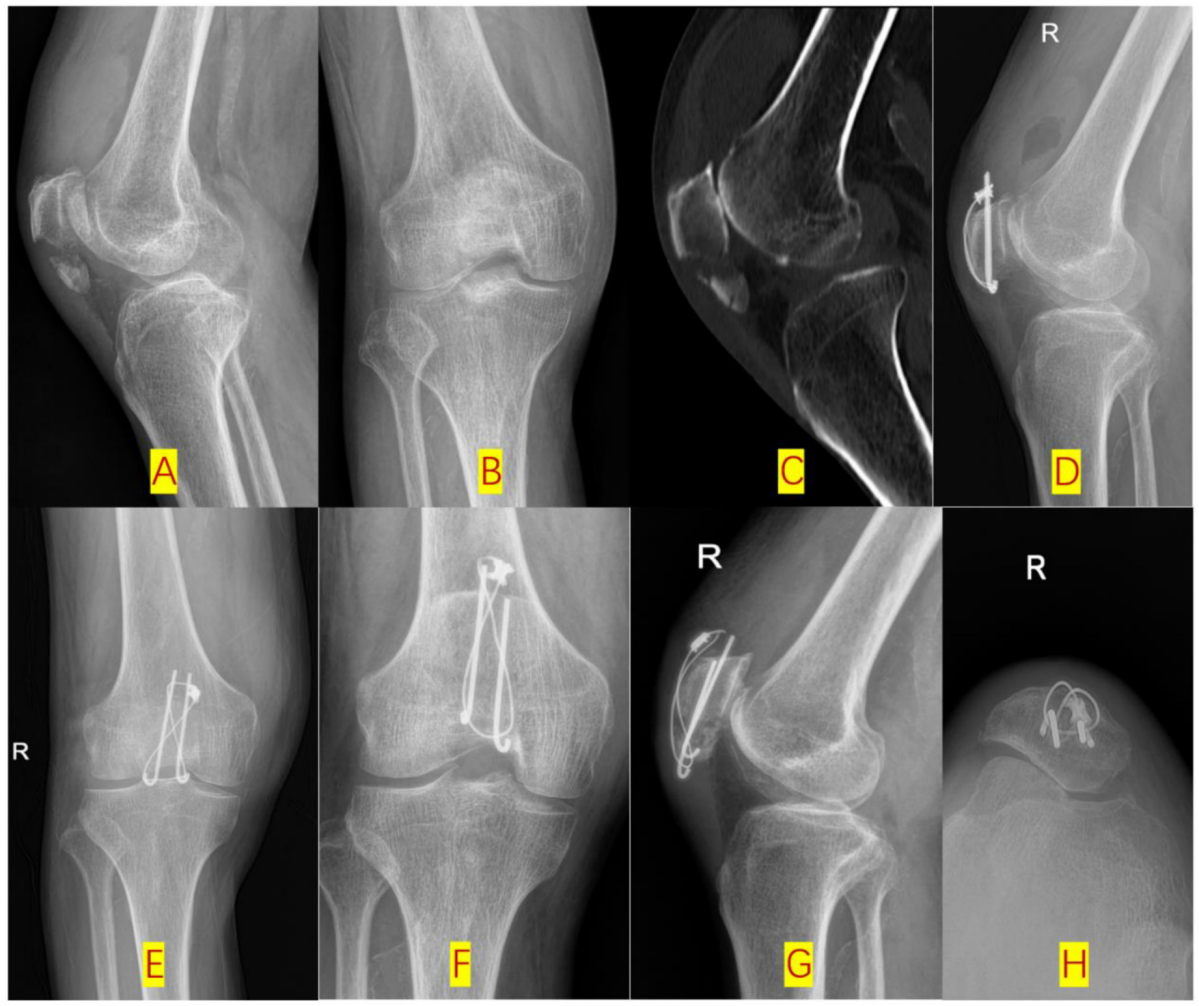
One case of internal fixation failure was observed in the KWC group at the 6-month postoperative follow-up **(A,B)**: pre-op x-ray lateral and PA views; **(C)**: pre-op CT sagittal reconstruction; **(D,E)**: 3-month post-op lateral and PA views; **(F–H)**: 6-month post-op hardware failure).

Complications: The incidence of symptomatic hardware irritation was significantly lower in the DCSC group (3.8% vs. 21.8%, *p* = 0.03). The incidence rates of other complications, including superficial infection (3.8% vs. 2.0%, *p* = 0.58), did not differ significantly between the groups. All patients achieved satisfactory healing, with no instances of nonunion or delayed union observed. At the 6-month postoperative follow-up, only one case of internal fixation failure was observed in the KWC group, where the cable and Kirschner wire became detached at the proximal patella. However, since the fracture had already fully healed, this did not lead to any adverse clinical outcomes (Figure 5).

Reoperation rates: There was no significant difference in the overall reoperation rate between the DCSC group and the KWC group (3.8% vs. 2.0%, *p* = 0.82).

### 3.3 Subgroup analysis

Compared with KWC fixation, the DCSC system demonstrated significant improvements in functional outcomes across all fracture classifications (C1, C2, C3), with the most pronounced benefits observed in C2 fractures (OTA 34-C2).

#### 3.3.1 Lysholm scores

C2 Fractures: At 3 months post-surgery, the DCSC group had a mean Lysholm score of 76.4 (SD 4.9), which was significantly greater than that of the KWC group (67.5 ± 2.6; mean difference = 8.9, *p* < 0.001). At 12 months, the DCSC group still had a significantly greater Lysholm score of 86.7 (SD 6.3) than the KWC group (91.2 ± 6.2; mean difference = −4.5, *p* = 0.046).

C1 and C3 Fractures: The DCSC system also showed significant advantages for C1 and C3 fractures at 3 months (C1: 75.2 vs. 71.2, *p* = 0.007; C3: 76.5 vs. 69.8, *p* = 0.001), but these differences diminished at 12 months (C1: 89.6 vs. 89.7, *p* = 0.961; C3: 89.5 vs. 89.9, *p* = 0.890) (Table 2).

**Table 2:** Comparison of Lysholm scores between the DCSC and KWC groups, stratified by fracture classification.

Classification	Time point (months)	DCSC mean (SD)	KWC mean (SD)	*t*	*p*
C1	3	75.2 (6.8)	71.2 (3.1)	2.83	0.007
	12	89.6 (6.7)	89.7 (6.0)	−0.05	0.961
C2	3	76.4 (4.9)	67.5 (2.6)	5.47	<0.001
	12	86.7 (6.3)	91.2 (6.2)	−2.05	0.046
C3	3	76.5 (7.4)	69.8 (2.9)	3.63	0.001
	12	89.5 (6.7)	89.9 (5.8)	−0.14	0.890

SD, standard deviation.

#### 3.3.2 Böstman scores

C2 Fractures: At 3 months post-surgery, the DCSC group exhibited the largest improvements (21.6 ± 0.5 vs. 17.2 ± 0.5; *p* < 0.001); this superiority was also observed at 12 months (22.1 ± 0.7 vs. 22.0 ± 0.9; *p* = 0.669). C1 and C3 Fractures: The DCSC system also showed significant advantages for C1 and C3 fractures at 3 months (C1: 21.5 vs. 17.5, *p* < 0.001; C3: 21.3 vs. 17.6, *p* < 0.001), but no differences were observed at 12 months (C1: 22.0 vs. 22.0, *p* = 0.933; C3: 22.0 vs. 22.0, *p* = 0.918).

In the PSM-matched cohort, C2 fractures (*n* = 10 per group) showed consistent results: DCSC had higher 3-month Lysholm scores (76.4 ± 4.9 vs. 67.1 ± 3.0, *p* < 0.001) but comparable 12-month scores (86.7 ± 6.3 vs. 90.9 ± 6.4, *p* = 0.09). The loss of significance in 12-month Lysholm scores (86.7 vs. 91.2, *p* = 0.046) may reflect limited statistical power (*post hoc* power = 35%) rather than clinical inferiority. The small sample size (*n* = 10 DCSC-C2) limits definitive conclusions (Table 3).

**Table 3:** Comparison of Böstman scores between the DCSC and KWC groups, stratified by fracture classification.

Classification	Time point (months)	DCSC mean (SD)	KWC mean (SD)	*t*	*p*
C1	3	21.5 (0.5)	17.51 (0.6)	22.3	<0.001
	12	22.0 (0.9)	21.98 (0.8)	0.08	0.933
C2	3	21.6 (0.5)	17.2 (0.5)	23.0	<0.001
	12	22.1 (0.7)	22.0 (0.9)	0.4	0.669
C3	3	21.3 (0.5)	17.6 (0.5)	16.3	<0.001
	12	22.0 (0.5)	22.0 (0.8)	−0.1	0.918

SD, standard deviation.

### 3.4 Surgical parameters

Compared with the KWC group, the DCSC group had a shorter mean operative time (62.9 ± 1.8 min vs. 76.0 ± 1.4 min, *p* < 0.001). However, there was no significant difference in the number of fluoroscopy shots between the two groups (*p* = 0.98).

### 3.5 Propensity score matching

After PSM, 26 patients from the DCSC group were successfully matched with 26 patients from the KWC group. Baseline characteristics were well-balanced between the matched groups (all SMD <0.1; Table 4). In the matched cohort, the DCSC group maintained significantly better outcomes at 3 months: Lysholm scores (76.0 ± 6.1 vs. 68.3 ± 3.1, *p* < 0.001), Böstman scores (21.5 ± 0.5 vs. 17.3 ± 0.6, *p* < 0.001), and lower hardware irritation rates (3.8% vs. 23.1%, *p* = 0.03). The 12-month functional scores and reoperation rates remained comparable between groups (*p* > 0.05).

**Table 4:** Baseline characteristics before and after propensity score matching.

Variable	Before PSM		After PSM		SMD
	DCSC	KWC	DCSC	KWC	
Age (years)	62.6 ± 13.5	60.0 ± 13.0	62.6 ± 13.5	61.2 ± 12.8	0.05
Male sex	20.5%	79.5%	20.5%	19.2%	0.02
Fracture C2	22.2%	77.8%	22.2%	23.1%	0.01

SMD, standardized mean difference.

## 4 Discussion

### 4.1 Key findings

This study aimed to compare the clinical efficacy and safety of the dual cannulated screw-cable (DCSC) system with the conventional Kirschner wire-cable (KWC) fixation in the management of patellar fractures, particularly focusing on functional recovery, radiographic union, and complication rates. The rationale for this comparison stems from the known limitations of KWC fixation, such as high rates of hardware irritation and loss of reduction, and the theoretical advantages of DCSC, including active interfragmentary compression and reduced soft tissue irritation. Our findings demonstrate that the DCSC system provides superior early functional recovery, as evidenced by significantly higher Lysholm and Böstman scores at 3 months postoperatively, particularly in complex intra-articular fractures (OTA 34-C2). Additionally, the DCSC group experienced shorter operative times, reduced symptomatic hardware irritation, and faster radiographic union compared to the KWC group. The superior outcomes in C2 fractures may reflect the DCSC system's ability to address metaphyseal comminution through interfragmentary compression, whereas C3 fractures may require additional stabilization beyond screw-cable constructs. However, the long-term functional outcomes at 12 months were similar between the two groups, suggesting that the benefits of DCSC diminish over time for simpler or more comminuted fracture patterns (C1/C3). The main conclusion of this study is that the DCSC system is a superior option for managing moderately complex patellar fractures (OTA 34-C2), associated with improved early recovery and fewer reoperations, while its incremental benefits in simpler fractures may not justify its routine use over KWC fixation.

Superiority in C2 Fractures: The DCSC system achieved a better early functional recovery in C2 fractures, as evidenced by both the Lysholm and Böstman scores. This finding highlights the biomechanical efficacy of the DCSC system in terms of managing early recovery of complex intra-articular fractures (OTA 34-C2), where interfragmentary compression and stable fixation are critical. However, the small sample size (*n* = 10 DCSC-C2) limits definitive conclusions.

Transient Benefits in C1/C3: While the DCSC system showed early advantages in C1 and C3 fractures, the long-term outcomes were not significantly different between the DCSC and KWC groups, suggesting that simpler fracture patterns or more comminuted fractures may not require enhanced compression between fragments provided by DCSC.

Clinical Implications These findings indicate that DCSC should be the preferred technique for C2 fractures, as its biomechanical advantages (e.g., active interfragmentary compression) translate to superior early and sustained functional outcomes. For C1 and C3 fractures, the DCSC system remains a viable option, but its incremental complexity over KWC may not be justified in routine cases.

Compared with conventional KWC fixation, the DCSC system demonstrated superior clinical outcomes, particularly in complex intra-articular fractures (OTA 34-C2). At 3 months post-surgery, the DCSC system achieved significantly higher Lysholm scores across all fracture classifications (C1: 75.2 vs. 71.2, *p* = 0.007; C2: 76.4 vs. 67.5, *p* < 0.001; C3: 76.5 vs. 69.8, *p* = 0.001), with the most pronounced improvement observed in C2 fractures (mean difference = 8.9, *p* < 0.001). Similarly, the Böstman scores at 3 months were markedly higher in the DCSC group (C1: 21.5 vs. 17.5; C2: 21.6 vs. 17.2; C3: 21.3 vs. 17.6; all *p* < 0.001). These benefits are attributed to the biomechanical superiority of active interfragmentary compression, which generates 41% greater compressive forces than does K-wire neutralization (5), thereby effectively minimizing fracture gap formation during early knee flexion (9). Cadaveric studies confirm cannulated screws withstand 41% greater cyclic loading than K-wires (5, 6), reducing gap formation during early flexion. Conversely, KWC offers flexibility in comminuted fractures but risks wire migration (3, 4).

However, the learning curve associated with DCSC implantation remains a challenge, particularly during the initial adoption phase. Nevertheless, the DCSC group demonstrated significantly shorter operative times than the KWC group (62.9 ± 1.8 min vs. 76.0 ± 1.4 min, *p* < 0.001). This difference is likely attributable to the streamlined surgical technique of DCSC, which eliminates the need for repeated fluoroscopic checks to confirm K-wire tip positioning and avoids the labor-intensive process of bending and securing K-wire ends. Nevertheless, early cases were associated with a 21% incidence of screw malalignment, as reported by Hoshino et al. (10), thus underscoring the importance of standardized training protocols to mitigate technical challenges and optimize surgical precision. Our findings align with a recent biomechanical study, W. J. Tee et al. (11) demonstrated that, compared with KWC, DCSC systems reduce soft tissue irritation by 78%, which is attributed to the low-profile screw design. Recent randomized trials comparing tension-band variations [e.g., Fiber Wire® (Smith & Nephew) vs. titanium cable (12)] report 32% lower irritation rates with polymer cables (*p* = 0.02) (13). Mittal S et al. (14) showed that conventional fixation techniques offer good union rates but at a cost of a high incidence of removal (13). Recent RCTs support reduced hardware irritation with polymer cables (12), though screw-based systems like DCSC retain superior compression stability (6, 13). Our findings align with Tee et al. (11), confirming DCSC's biomechanical advantage in minimizing soft tissue irritation.

### 4.2 Limitations

First, although PSM balanced measurable confounders, residual selection bias may persist due to unadjusted factors (e.g., surgeon experience, subtle fracture complexity variations). The DCSC group was operated exclusively by two surgeons proficient in this technique, while the KWC group was managed by surgeons without DCSC experience. This introduces performance bias that cannot be fully eliminated by statistical adjustment. The learning curve for DCSC implantation—reflected in a 21% screw malalignment rate during early adoption (10)—may have influenced outcomes. We mitigated this through standardized training protocols after 2022, including: (1) Preoperative 3D fracture mapping, (2) Intraoperative navigation guides for screw trajectory, and (3) Mentored proctoring for initial 10 cases. Despite these measures, the technical complexity of DCSC remains a barrier for surgeons transitioning from KWC.

Although PSM adjusted for measurable confounders, unmeasured factors (e.g., subtle fracture complexity variations) may persist. The learning curve for DCSC—reflected in early screw malalignment rates (10)—was mitigated by standardized protocols after 2022. Although the DCSC group showed superior early functional recovery in C2 fractures, the marginally higher Lysholm score in the KWC group at 12 months (91.2 vs. 86.7, *p* = 0.046) should be interpreted with caution. This reversal may stem from the small sample size of the DCSC C2 subgroup (*n* = 10), where minor variations could amplify statistical differences. Caution is warranted in interpreting C2 subgroup results due to the small cohort; larger studies are needed to validate these findings. Additionally, as both groups achieved near-normal knee function by 12 months (Lysholm >85), the clinical relevance of this statistical difference is likely negligible. Long-term studies with balanced subgroups are needed to validate these findings.

Second, unmeasured confounders, such as surgeon experience, rehabilitation compliance, and patient-specific factors (e.g., bone quality), were not fully adjusted in the analyses. These factors could influence functional recovery and complication rates, potentially introducing bias. Nevertheless, the significant improvements in early functional outcomes and reduced hardware irritation in the DCSC group are consistent with the biomechanical advantages of the system, supporting the validity of the findings.

Third, the median follow-up period of 12 months limits the ability to assess long-term complications, such as screw loosening in osteoporotic bone or late hardware failure. While the study provides valuable insights into early and mid-term outcomes, longer follow-up durations are necessary to evaluate the durability of the DCSC system, particularly in patients with compromised bone quality. Future studies should prioritize extended follow-up periods (>5 years) to address these concerns and provide a more comprehensive understanding of the long-term performance of DCSC fixation.

Despite these limitations, the study's findings remain robust, as they are supported by significant improvements in functional scores, reduced complication rates, and faster radiographic union in the DCSC group. These results align with previous biomechanical studies and clinical reports, reinforcing the credibility of the conclusions. To further validate these findings, future research should focus on multicenter randomized controlled trials with standardized surgical protocols and longer follow-up durations (15). Additionally, integrating emerging technologies, such as 3D-printed guides and bioabsorbable cables, could help optimize surgical precision and outcomes, addressing some of the technical challenges associated with DCSC implantation.

### 4.3 Clinical implications

Based on the current evidence, the findings of this study have several important clinical implications for the management of patellar fractures. The DCSC system demonstrated superior early functional recovery, reduced symptomatic hardware irritation, and faster radiographic union compared to the conventional KWC fixation, particularly in complex intra-articular fractures (OTA 34-C2). These benefits are attributed to the biomechanical advantages of the DCSC system, including active interfragmentary compression and a low-profile design that minimizes soft tissue irritation. Therefore, we recommend DCSC as the first-line treatment for comminuted patellar fractures (OTA 34-C2), as its biomechanical stability and reduced complication profile justify its application in these complex cases. While C3 fractures are typically more comminuted, C2 fractures in our cohort involved less significant metaphyseal comminution with articular displacement, yet the latter still posed notable challenges due to the need for precise articular reduction.

However, for simpler or more comminuted fracture patterns (C1/C3), the long-term functional outcomes were comparable between the DCSC and KWC groups, suggesting that the incremental complexity of the DCSC system may not be justified in routine cases. In these scenarios, KWC fixation remains a viable option, particularly in settings where surgical expertise in DCSC is limited or when cost and resource considerations are a concern. The transient benefits of DCSC in simpler or more comminuted fracture patterns (C1/C3) highlight the importance of tailoring the surgical approach to the specific fracture type and patient needs.

The learning curve associated with DCSC implantation, particularly during the initial adoption phase, should also be considered. Early cases may be associated with technical challenges, such as screw malalignment, as reported in previous studies. Therefore, standardized training protocols and surgical guides are essential to optimize surgical precision and minimize complications. The shorter operative times observed in the DCSC group, despite the learning curve, suggest that once proficiency is achieved, the technique can be efficiently integrated into clinical practice.

Future research should prioritize multicenter randomized controlled trials with extended follow-up durations (>5 years) to further validate the long-term durability and efficacy of the DCSC system, particularly in patients with osteoporotic bone or other comorbidities. Additionally, integrating emerging technologies, such as 3D-printed guides and bioabsorbable cables (16, 17), could enhance surgical precision and reduce complications, further improving outcomes in patellar fracture management.

In summary, the DCSC system offers significant advantages for complex patellar fractures (OTA 34-C2), making it the preferred technique in these cases. For simpler or more comminuted fracture patterns (C1/C3), KWC fixation remains a viable option, and the choice of fixation method should be guided by fracture complexity, surgeon experience, and patient-specific factors. Future studies should focus on refining surgical techniques and exploring advanced technologies to optimize outcomes and address the limitations identified in this study.

## 5 Conclusion

### 5.1 Key findings

The dual cannulated screw-cable (DCSC) system demonstrates superior early outcomes vs. Kirschner wire-cable (KWC) fixation for patellar fractures, particularly in complex intra-articular fractures (OTA 34-C2).

### 5.2 Clinical implications

DCSC is preferred for C2 fractures when early mobility is critical due to its biomechanical advantages (active interfragmentary compression, low-profile design), but surgeon experience and fracture pattern must guide selection. For simpler (C1) or more comminuted (C3) fractures, DCSC's benefits diminish by 12 months (Lysholm/Böstman *p* > 0.05), supporting KWC as a viable alternative when resources/expertise are limited. Technical caution: Avoid DCSC in distal coronal plane comminution due to compression limitations.

### 5.3 Research gaps

Long-term durability (>5 years), especially in osteoporosis, and integration of 3D-printed guides/bioabsorbable cables warrant further multicenter trials.

## Data Availability

The original contributions presented in the study are included in the article/Supplementary Material, further inquiries can be directed to the corresponding author.
