# Supplementary material for: Comparative clinical outcomes of dual cannulated screw-cable system vs. Kirschner wire-cable fixation in type C patellar fractures
Source: Front Surg. 2025 Sep 1;12:1594907. doi: 10.3389/fsurg.2025.1594907 (PMC12439163; doi:10.3389/fsurg.2025.1594907)
Supplement: Supplementary file 1 [file Datasheet1.docx]

*Suppl. Table 1* Raw Data Summary Table

| Fracture Type | DCSC(n=26) | KWC(n=101) | Total(n=127) | Follow-up completion |
| --- | --- | --- | --- | --- |
| OTA 34-C1 | 10（38.5%） | 41（40.1%） | 51 | 51/51（100%） |
| OTA 34-C2 | 10（38.5%） | 35（34.7%） | 45 | 45/45（100%） |
| OTA 34-C3 | 6（23.1%） | 25（24.8%） | 31 | 31/31（100%） |
| PSM-matched C2 sub-group | 10 | 10 | 20 | 20/20（100%） |

*Subgroup Distribution & Follow-up Completion*

*Suppl. Figure 1* CONSORT-style flow diagram of patient enrollment and matching


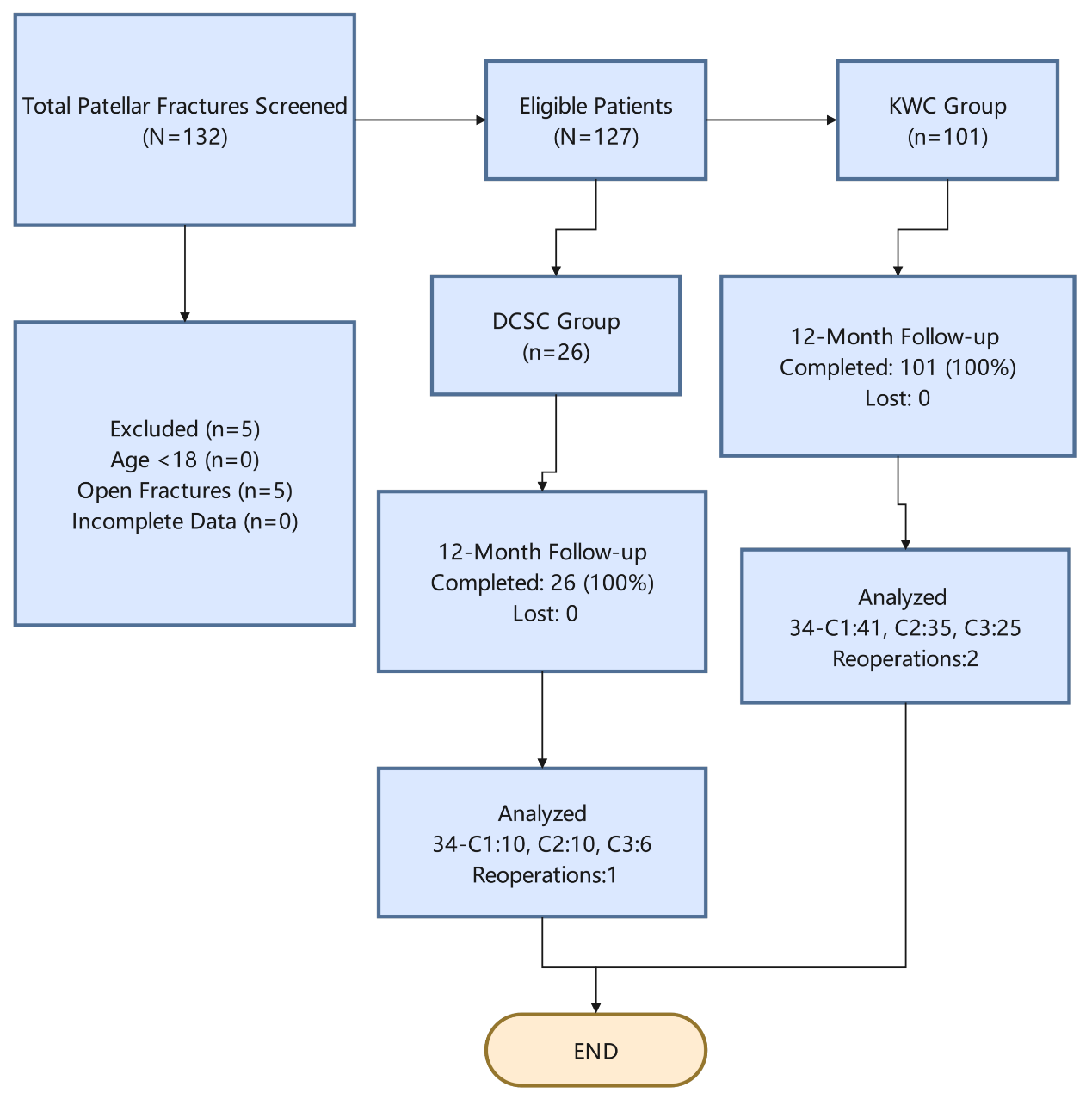


*CONSORT flow diagram of patient screening, allocation, and follow-up in this retrospective cohort study.*

The data utilized in this study are publicly available in mandated datasets on Figshare.com, accessible through the link DOI: 10.6084/m9.figshare.28797593.
